# Genetic and Epigenetic Association of *FOXP3* with Papillary Thyroid Cancer Predisposition

**DOI:** 10.3390/ijms25137161

**Published:** 2024-06-28

**Authors:** Charoula Achilla, Angeliki Chorti, Theodosios Papavramidis, Lefteris Angelis, Anthoula Chatzikyriakidou

**Affiliations:** 1 Laboratory of Medical Biology and Genetics, Faculty of Medicine, School of Health Sciences, Aristotle University of Thessaloniki, 54124 Thessaloniki, Greece; cachilla@auth.gr; 2 First Propedeutic Department of Surgery, AHEPA University Hospital, Aristotle University of Thessaloniki, 54124 Thessaloniki, Greece; chorange2404@gmail.com (A.C.); tpapavra@auth.gr (T.P.); 3 School of Informatics, Aristotle University of Thessaloniki, 54124 Thessaloniki, Greece; lef@csd.auth.gr

**Keywords:** *FOXP3*, *PPP1R3F*, genetic association, epigenetic association, genetic variant, polymorphism, methylation, papillary thyroid cancer

## Abstract

Papillary thyroid cancer (PTC) is the most common type of thyroid malignancy with an increased female incidence ratio. The specific traits of X chromosome inheritance may be implicated in gender differences of PTC predisposition. The aim of this study was to investigate the association of two X-linked genes, Forkhead Box P3 (*FOXP3*) and Protein Phosphatase 1 Regulatory Subunit 3F (*PPP1R3F*), with PTC predisposition and gender disparity. One hundred thirty-six patients with PTC and an equal number of matched healthy volunteers were enrolled in the study. Genotyping for rs3761548 (*FOXP3*) and rs5953283 (*PPP1R3F*) was performed using polymerase chain reaction–restriction fragment length polymorphism assay (PCR-RFLP). The methylation status of *FOXP3* was assessed using the combined bisulfite restriction analysis (COBRA) method. The SPSS software was used for statistical analyses. Gender stratification analysis revealed that the CA and AA genotypes and the A allele of *FOXP3* rs3761548 variant are associated with PTC predisposition only in females. Moreover, different methylation status was observed up to the promoter locus of *FOXP3* between PTC female patients, carrying the CA and CC genotype, and controls. Both revealed associations may explain the higher PTC incidence in females through reducing FOXP3 expression as reported in immune related blood cells.

## 1. Introduction

Thyroid cancer (TC) is the most prevalent endocrine malignancy, representing 3.4% of all cancers diagnosed annually worldwide. Its incidence has been increasing rapidly over the last decades due to improvements in detection methods which lead to earlier diagnosis of the disease [1,2]. Thyroid tumors are classified into five histological types: papillary TC (PTC), follicular TC (FTC), poorly differentiated TC (PDTC), medullary TC (MTC), and anaplastic TC (ATC) [1]. PTC is the most common type, accounting for approximately 80% of all TC cases [3]. Exposure to ionizing radiation, alcohol and tobacco, obesity, and family history are considered risk factors for TC [2].

Moreover, genetic factors are implicated in TC susceptibility. TC is often caused by chromosomal rearrangements on the *RET* gene or point mutations on the *RAS* and *BRAF* proto-oncogenes, *TP53* onco-suppressor gene, and *TERT* gene [3,4]. Moreover, genetic variants in ten genetic loci have been associated with TC susceptibility in genome-wide association studies (GWAS) [4,5], but these findings only partially explain the genetic basis of TC, as they do not appear in all TC patients. Furthermore, the X chromosome is underrepresented in GWAS because of the specific traits of X chromosome inheritance [6].

However, TC has a female-to-male incidence ratio of 2.75:1 and is the fifth most common cancer type in women [6]. Regarding the histologic subtype of TC, PTC is more common in women, while the more aggressive types such as ATC and MTC have similar gender distributions. Moreover, men have a worse survival rate and a more aggressive di-sease at diagnosis [7,8]. Although many studies have attempted to associate gender disparities in TC with environmental, hormonal, and molecular factors, the results are inconsistent, and the female PTC predominance remains unclear [8]. 

Taking into account the genomic differences between two genders, the X chromosome could have a role in the gender disparity observed in PTC susceptibility. In each somatic cell in females, one of the two X chromosomes is randomly submitted to an inactivation process through epigenetic modifications to equalize the gene-dosage effects of sex chromosomes. Defects of X inactivation have been reported in X-linked genes and related to diseases [9]. Various X-linked genes have been involved in tumorigenesis, and some of them have been reported to show altered expression profiles in TC [3,10]. Additionally, in a recent review of our group, genetic variants on fourteen X-linked genes have been reported to be involved in cancer predisposition in a sex-associated way [11]. Considering the specific traits of X chromosome inheritance and inactivation, it is important to investigate the possible role of X-linked genes in TC gender disparity.

The present candidate gene study focuses on the role of two X-linked genes, *FOXP3* (Forkhead Box P3) and the *PPP1R3F* (Protein Phosphatase 1 Regulatory Subunit 3F), in PTC predisposition based on a priori biological hypothesis due to the described role of the selected genes in cancer predisposition [12,13]. *FOXP3* (Xp11.23) encodes FOXP3 transcription factor, a member of the forkhead family of proteins, which regulates the deve-lopment and function of T regulatory cells. Altered expression of *FOXP3* has been observed in many autoimmune diseases and cancer types, including TC [14], with rs3761548 *FOXP3* polymorphism mainly associated with several studied cancer types [15,16]. *PPP1R3F* encodes a regulatory subunit of type-1 protein phosphatase (PP1) implicated in glycogen metabolism. Despite the widely recognized involvement of PP1 in key biological processes, the recent literature provides compelling evidence that supports major roles for PP1 in tumorigenesis [17]. In addition, hypermethylation of *PPP1R3F* has been associated with colorectal cancer risk [18], and rs5953283 *PPP1R3F* polymorphism has been linked to asthma [19]. Therefore, the aim of our study is to investigate the genetic and functional association of *FOXP3* rs3761548 (C > A, in promoter) [20,21] and *PPP1R3F* rs5953283 (A > G, in intron) [19] variants in PTC susceptibility and gender disparity.

## 2. Results

*FOXP3* rs3761548 and *PPP1R3F* rs5953283 genetic polymorphisms were in Hardy–Weinberg equilibrium in female control group (*p* = 0.49, *p* = 0.37; respectively). The two studied genetic variants in the patient (r^2^ = 0.41, D’ = 0.79) and control (r^2^ = 0.26, D’ = 0.67) groups show moderate linkage disequilibrium. Taking into account that the female patient and control groups had a size of 100 participants, respectively, we achieved a power of 80% and a level of significance of 5% (two sided) for detecting an effect size of 0.40 between pairs. In addition, since male patient and control groups had a size of 36 subjects, respectively, we achieved a power of 80% and a level of significance of 5% (two-sided), for detecting an effect size of 0.67 between pairs. The above studied groups show medium and large effect sizes, respectively, according the standards for interpretation in medical research [22], which indicate the practical significance of the research outcomes.

Strong statistically significant differences were detected in *FOXP3* rs3761548 genotype distributions between female PTC patients and controls according to the additive, homozygous, heterozygous, and dominant model (*p* = 0.03, *p* = 0.01, *p* = 0.02, *p* = 0.01; respectively). Moreover, the rs3761548 A allele was revealed as the risky variant in females (*p* = 0.02, OR: 1.58, 95% CI: 1.07–2.36), but not in males (*p* = 0.63, OR: 1.25, 95% CI: 0.5–3.15). When we studied the allelic distribution in the whole population, the rs3761548 A allele showed statistically significant association with PTC (*p* = 0.02), but this may be attributed to the strong effect of the 2.75-fold larger number of females compared to males. Regarding *PPP1R3F* rs5953283 variant, no statistically significant difference was observed in the distribution of genotypes or alleles between PTC patients and controls. Results of all genetic association analyses of rs3761548 and rs5953283 with PTC are shown in Table 1.

**Figure 1:**
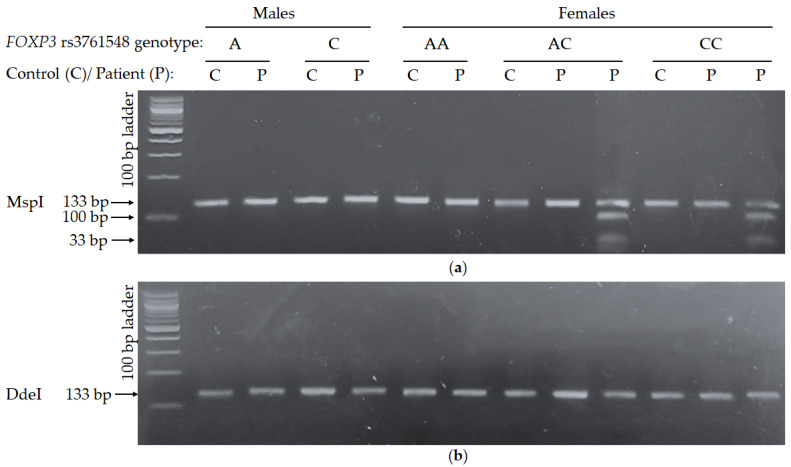
*FOXP3* methylation status observed in relation to rs3761548 genotypes in female and male PTC patients and controls. (**a**) MspI digestion reveals the methylation status upstream the *FOXP3* promoter region, (**b**) DdeI digestion confirms the complete DNA sodium bisulfite conversion of samples.

In addition, the Combined Bisulfite Restriction Analysis (COBRA) method was performed on twenty-five PTC patients and twenty-five controls, with respect to their rs3761548 genotypes, to assess the methylation status of CpG islands next to the *FOXP3* promoter region. All control, male, and female samples were unmethylated in the studied region. Regarding patients, all male patients’ samples were found to be unmethylated, while positive methylation status was detected in five out of fifteen female patients’ samples (Table 2). Statistically significant difference was observed in methylation status bet-ween female PTC patients vs. female controls (*n* = 5/15 vs. 0/15, *p* = 0.02). The methylation status in combination with rs3761548 genotypes revealed that methylated cytosines were detected only in female patients carrying the AC or CC genotypes and the pattern of po-sitive methylation was in heterozygosity (Table 2, Figure 1).

## 3. Discussion

TC is the most common endocrine malignancy and one of the most rapidly increasing types of cancer worldwide [2]. Understanding the genetic basis of TC may have potential diagnostic, prognostic, and therapeutic significance for the management of the disease. The present study aims to the identification of genetic and epigenetic DNA risk factors which may be implicated in PTC predisposition and might be used for optimization of PTC prognosis and diagnosis in the future. Specifically, this study focuses on the role of *FOXP3* in PTC susceptibility and gender disparity.

*FOXP3* encodes a transcriptional regulator implicated in the function of T-regulatory cells. *FOXP3* is reported to be implicated in tumorigenesis in many cancer types acting either as a transcriptional activator or tumor-suppressor gene [14]. Our results indicated that the A allele and the CA and AA genotypes of rs3761548 genetic variant, located in the promoter region of *FOXP3*, showed strong statistically significant difference in their distribution between female PTC patients and controls. Our findings are consistent with a previous study which indicated an association of the CA and AA genotypes of rs3761548 genotypes with differentiated TC (DTC) risk in a Han Chinese population. The frequency of these genotypes was also higher in female compared to male DTC patients [23]. This may partially explain the increased prevalence of PTC in females compared to males. Association between the A allele of rs3761548 variant and disease risk only in females has also been reported for multiple sclerosis and Graves’ disease, and for breast cancer progression [24,25,26,27]. Thus, rs3761548 may be implicated in the genetic basis of the diseases in a sex-associated way.

Independently from sex, association between the rs3761548 A allele or AA genotype with tumorigenesis has also been reported for colorectal cancer, gastric adenocarcinoma, and lung cancer predisposition [28,29,30]. On the other hand, the C allele of rs3761548 has been detected to increase oral cancer and endometrial cancer susceptibility [31,32]. These contradictory findings could be explained by differences in the associated alleles in expression of FOΧP3 in T cells or tumor cells that can have different impacts on cancer tumor microenvironment and cancer outcomes [33].

Moreover, our research was expanded to the methylation status of a CpG islands next to the promoter region of *FOXP3*. Several studies have highlighted a correlation between differentially methylated regions near promoter regions and gene expression changes [34]. DNA methylation is one of the epigenetic mechanisms that regulate genome inte-grity, gene expression, and X-inactivation. Hypermethylation of CpG islands in the promoter region or gene body reduces gene expression and has been associated with many cancer types [35]. According to a previous study, *FOXP3* generally lack CpG islands [36], while demethylation in the *FOXP3* locus is associated with increased *FOXP3* expression and Treg stability [37,38]. The results of the present study revealed that there was no me-thylation in male or female controls independently of their rs3761548 genotypes and in PTC patients for all males carrying the A or C genotype but for females only for those carrying the AA genotype. In contrast, in some female PTC patients carrying the CC or CA genotypes, methylated cytosines were detected, indicating a possible interplay bet-ween *FOXP3* methylation status and rs3761548 genotypes in PTC predisposition.

Our study focused on a CpG region next to the promoter of *FOXP3*, which is also next to the promoter of *PPP1R3F*, since these genes are mapped in DNA strands which share the same intergenic region between their promoters (*FOXP3*-reverse strand, *PPP1R3F*-forward strand). To validate the revealed association of rs3761548 variant and *FOXP3* methylation status with PTC, we performed genotypic analysis of rs5953283 *PPP1R3F* polymorphism, previously related to asthma [19], in order to examine if this variant and gene, due to linkage disequilibrium (LD), misled to the revealed *FOXP3* association with PTC predisposition. According to 1000 Genome data for the two studied SNPs, *FOXP3* rs3761548 and *PPP1R3F* rs5953283, in all population and specifically in Caucasians (CEU) are in strong LD (All: r^2^ = 0.3242, D’ = 0.9956; CEU: r^2^ = 0.4669, D’ = 1) [39]. *PPP1R3F* encodes one of the protein–phosphatase-1 catalytic subunits involved in glycogen meta-bolism, which has previously been associated with colorectal cancer [18]. *PPP1R3F* has been reported to have CpG islands in contrast to *FOXP3*, which could also mean different potential in their methylation status [36]. However, no statistical difference was observed in the prevalence of rs5953283 *PPP1R3F* genotypes between PTC patients and controls. The LD study of rs3761548 and rs5953283 in our patient and control samples show that it is moderate, which may excuse why only the rs3761548 variant of *FOXP3* has been associated with PTC. All the above could strengthen the validity of the revealed genetic and epigenetic associations of *FOXP3* with PTC risk.

The combined analysis of *FOXP3* rs3761548 genotypes and methylation status revealed that methylated cytosines were detected only in female patients carrying a rs3761548 C allele. Thus, we could assume that the C allele drives methylation in female patients with CA or CC genotype. In contrast, in healthy individuals carrying the CA or CC genotype, both alleles were unmethylated. The AA genotype of rs3761548 was also previously associated with defective transcription of *FOXP3,* since the rs3761548 A allele has been shown to disrupt transcription factor binding [40,41]. In addition, the A allele has been reported to reduce *FOXP3* expression and affect Treg function and immunomo-dulatory cytokine secretion [29,42,43]. Thus, in the present study, reduced expression of *FOXP3* in PTC patients relative to their rs3761548 genotypes can be attributed either to the presence of A allele or to the methylation of C allele. Moreover, taking into account that the positive methylation was observed only in female patients, this may explain the increased incidence of PTC in females vs. males.

In recent years, an increasing amount of evidence points to the significance of using DNA methylation in peripheral blood as a potential diagnostic biomarker for cancer and other diseases [44,45,46,47,48,49]. Variant rs3761548 has been associated with a variety of cancer types [23,28,30,31,32] and autoimmune diseases [43,50,51,52]. Considering the results of our study, it is important to analyze *FOXP3* DNA methylation status in other diseases, which have rs3761548 implicated in their pathogenesis, in order to identify the role of the interaction of DNA methylation and rs3761548 in these diseases’ risk.

To our knowledge, this is the first study which correlates the rs3761548 *FOXP3* va-riant in combination with gene’s methylation status with PTC predisposition. Since both the rs3761548 A allele and methylated C allele potentially decrease *FOXP3* expression, this could mean lower numbers of the FOXP3+ regulatory T (Treg) cells which normally express it [https://www.proteinatlas.org/ENSG00000049768-FOXP3/immune+cell (accessed on 16 April 2024)] It is known that Treg cells expressing the transcription factor FOXP3 have a critical role in the maintenance of immune homeostasis and prevention of autoimmune and inflammatory diseases [53,54]. Reduced levels of FOXP3+ Treg cell have been associated with autoimmune and inflammatory diseases [55]. It is commonly accepted that thyroid cancer is linked to inflammation [56,57], which may explain the genetic and epigenetic associations of the present study with PTC predisposition, both of which could lead to reduced *FOXP3* expression in blood cells, as other authors also reported for rs3761548 A allele [41,58,59].

However, the inconsistencies of other studies, which report increased *FOXP3* expression in PTC patients, may be attributed to differences of FOXP3 isoforms expressed among different cell types, FOXP3 subcellular localization, the studied Treg and tumor cell types, the methods of analyses, the sizes of compared samples, the tumor location and stage to draw conclusions, while in the vast majority of studies of FOXP3 expression they have not been conducted in association with *FOXP3* rs3761548 genotypic data [23,53,60,61,62,63,64,65,66]. Probably, the observed upregulation of FOXP3 in thyroid cancer cell is just the infiltration of FOXP3+ Treg cells in tumor microenvironment surveillance against cancer [67], which does not necessary reflect an increase of FOXP3 in Treg cells. Conversely, reduced expression of *FOXP3* in blood cells was reported in carriers of the associated *FOXP3* rs3761548 A allele with PTC predisposition [41,58,59].

## 4. Materials and Methods

### 4.1. Study Subjects

One hundred thirty-six unrelated patients with PTC (36 males, mean age 49 ± 13 years) were enrolled in the study, which coincides with the reported 2.75-fold higher incidence of female vs. male PCT susceptibility. All patients had newly diagnosed PTC staged as T1 N0 M0 or T2 N0 M0 and underwent total thyroidectomy between November 2022 and December 2023 in the 1st Propaedeutic Department of Surgery, AHEPA University Hospital of Thessaloniki, Greece. Patients were diagnosed according to the criteria of the American Cancer Society and had a PTC of less than 4 cm that was confined into the thyroid gland (no microscopic or macroscopic extra-thyroidal extension). In addition, they had no apparent lymph node spread (clinical or imaging negative lymph nodes).

An equal number of ethnic-, age- and sex-matched healthy volunteers with no personal or family history of neoplasia, or chronic autoimmune or infectious diseases were used as controls (36 males, mean age 50 ± 18 years). Both female and male control subjects and patients at the time of thyroidectomy had hormonal profiles within the normal refe-rence range for sex serum plasma hormones, such as follicle stimulating hormone (FSH), luteinizing hormone (LH), testosterone, estradiol, and progesterone [68], and for thyroid-related serum plasma hormones, such as thyroid stimulating hormone (TSH), free triiodothyronine (fT3), and free thyroxine (fT4) [69].

The study protocol was approved by the Ethics Committees of Aristotle University of Thessaloniki. A written informed consent was obtained from each patient before sample collection.

### 4.2. Genotype Analysis

Genomic DNA was extracted from peripheral blood lymphocytes using the PureLink Genomic DNA Kit (Invitrogen, Karlsruhe, Germany). All samples were genotyped for the studied genetic polymorphisms using polymerase chain reaction–restriction fragment length polymorphism (PCR-RFLP) assay. PCR reaction was carried out in a final volume of 25 mL containing 100 ng DNA, 2 µM of each primer (Integrated DNA technologies, Coralville, IA, USA), 2 U of OneTaq DNA polymerase (New England Biolabs GmBH, Frankfurt, Germany), 1x reaction buffer, 0.2 µM dNTP Mix (New England Biolabs GmBH, Frankfurt, Germany) and sterile deionized water. The primers and conditions used for PCR amplifications are shown in Table 3.

For rs3761548 genotype analysis, we followed the previously described methodology [70]. The amplicon size of the target sequence was 399 base pairs (bp) and after digestion with PstI restriction enzyme (New England Biolabs GmBH, Frankfurt, Germany), two fragments of 188 bp and 211 bp were obtained for the C allele, while the A allele remained uncleaved with 399 bp. Regarding rs5953283 variant, the amplicon size was 222 bp. DdeI restriction enzyme (New England Biolabs GmBH, Frankfurt, Germany) was used to digest PCR products, and genotypes were detected as follows: two fragments of 44 bp and 178 bp were obtained for the A allele, while the 222 bp PCR product remained uncleaved for the G allele. All samples were run twice.

### 4.3. Bioinformatic Prediction of CpG Islands Upstream the Promoter Region of FOXP3 Gene

To investigate the relationship between *FOXP3* expression and promoter methylation, 9035 base pairs of *FOXP3* promoter from the National Center for Biotechnology Information (NCBI) were analyzed by EMBOSS CpGplot software [https://www.ebi.ac.uk/Tools/seqstats/emboss_cpgplot/ (accessed on 10 December 2023)] to identify and plot CpG islands in nucleotide sequence [71]. *FOXP3* rs3761548 polymorphism, which was associated with PTC in the present study, is located at site 49,261,784, and the search for CpG islands was conducted in the region from 49,260,835 up to 49,269,870. We used the following criteria for CpG islands prediction: minimum CpG island length > 200 bp, CG content > 50% and obs/exp ratio > 0.6. CpG islands of *FOXP3*, with a length of 564 bp, were revealed in the locus from 49,269,252 up to 49,269,815.

### 4.4. Combined Bisulfite Restriction Analysis

MethPrimer 2.0 software was used to design PCR primers (Table 3) for the study of methylation status of the locus 49,269,252 up to 49,269,815 with a restriction analysis assay [https://www.urogene.org/methprimer/ (accessed on 10 December 2023)] [72]. DNA me-thylation status of CpG islands in the *FOXP3* promoter was assessed using the Combined Bisulfite Restriction Analysis (COBRA) methodology.

The methylation study was conducted in twenty-five PTC patients with specific *FOXP3* rs3761548 genotypes (5 males with A genotype, 5 males with C genotype, 5 females with CC genotype, 5 females with CA genotype, and 5 females with AA genotype) and an equal number of sex, age, and rs3761548 genotyping matched controls. Having studied 25 pairs of PTC cases and controls, our methylation study has over 85% power to detect a mean difference of 9% in methylation at nominal significance *p*-value ≤ 0.05 [73].

The extracted DNA from peripheral blood cells of the studied samples was treated with sodium bisulfite using the EpiTect Bisulfite Kit (Qiagen, Hilden, Germany) according to the manufacturer’s instructions.

Bisulfite-converted DNA was used as template for PCR amplification of a 133 bp region containing CpG islands next to the promoter of *FOXP3*. PCR amplification was carried out in a final volume of 25 µL containing 100 ng of converted DNA, 2 µM of each primer (Integrated DNA technologies, Coralville, IA, USA), 2 U of Platinum II Taq Hot-Start DNA Polymerase (Invitrogen, Karlsruhe, Germany), 1x reaction buffer, 0.2 µM dNTP Mix (New England Biolabs GmBH, Frankfurt, Germany) and sterile deionized water. The thermal conditions used in PCR reaction for COBRA analysis are shown in Table 3.

Then the PCR products were digested using the restriction enzyme MspI (New En-gland Biolabs GmBH, Frankfurt, Germany), which has a recognition site inside a CpG island in PCR products. In the case of methylated cytosines, the digestion of PCR product produces two fragments of 100 bp and 33 bp sizes, as the methylated CpG island, targeted by MspI restriction endonuclease, is retained after bisulfite conversion and the 133 bp PCR product is cleaved by MspI. In addition, a control digestion was performed for each studied sample with DdeI restriction enzyme (New England Biolabs GmBH, Frankfurt, Germany) to ensure the complete DNA sodium bisulfite conversion. The DdeI digestion sequence in PCR products is destroyed by the bisulfite conversion, so only PCR products with incomplete sodium bisulfite conversion are cleaved by DdeI, giving two fragments with sizes of 92 bp and 41 bp.

### 4.5. Statistical Analysis

Linkage disequilibrium analysis between rs3761548 and rs5953283 was conducted by calculating the squared correlation coefficient r^2^ and the standardized coefficient D’ using PLINK v1.9 software [74]. Pearson’s chi-square test was used to examine possible deviation from Hardy-Weinberg equilibrium (HWE) of the studied X-linked variants in control females. The effect sample sizes were calculated for male (*n* = 36) and female (*n* = 100) patient and control groups of comparisons in order to achieve a power of 80% and a level of significance of 5% (two sided) [75].

Differences in *FOXP3* rs3761548 and *PPP1R3F* rs5953283 distributions between PTC patients and controls were tested under the six models of genetic association, which are the homozygote, heterozygote, dominant, recessive, allelic and additive using Pearson’s chi-square test. Furthermore, the odds ratio (OR) with a confidence interval (CI) of 95% was calculated (reference allele vs. variant allele). One-tailed Fisher’s exact test was used to test the differences in methylation status between PTC patients and controls, as the expected values were less than 5. Considering the hemizygous state of X-linked genes in males and the X inactivation process in females, statistical analysis was performed by gender stratification to achieve correct type I error rate control and to address the complication of baseline allele specification which otherwise affects the association inference for an X-chromosomal SNP, in contrast to an autosomal SNP [76]. The SPSS Version 28 statistical package (IBM Corp., Armonk, NY, USA) was used for all statistical tests and a *p*-value less than 0.05 was considered as statistically significant.

## 5. Conclusions

Our candidate gene studies, the first in Caucasians, and another one in Chinese patients, agree that *FOXP3* rs3761548 A allele is associated with thyroid cancer predisposition [23]. Taking into account the relative allele frequency of rs3761548 A allele in different population groups according to 1000 Genome data [https://www.ensembl.org/Homo_sapiens/Info/Index (accessed on 20 June 2024)] with people of African origin to show the lowest frequency, this may also explain the results of the most recent study in which authors concluded that people of African descent had a lower risk of thyroid malignancy compared to other racial groups and that socioeconomic factors did not alter the racial disparity of thyroid cancer [77]. The *FOXP3* rs3761548 A allele was also revealed as risk factor in the majority of different cancer types, as described above, related to reduced FOXP3 expression in blood cells. To this association, we added, moreover, the positive association of *FOXP3* methylation status related to rs3761548 C allele which further explains the reduced FOXP3 expression in female carries of rs3761548 CA or CC genotypes. The latter may also excuse the increased incidence of PTC in females. Further independent studies in larger groups of patients of various ethnicities are needed to replicate and validate the present genetic and epigenetic associations of *FOXP3* rs3761548 genotypes and *FOXP3* methylation status to PTC risk and other cancer types.

## Figures and Tables

**Table 1:** Statistical analysis of rs3761548 and rs5953283 variants between PTC patients and controls.

Genotypes	Patients (*n* = 136)	Controls (*n* = 136)	Statistical Model	OR (95% CI) ^1^	*p*-Value
rs3761548					
Males (*n* = 36)					
C	17	19	Allelic (A vs. C)	1.25 (0.5–3.15)	0.63
A	19	17			
Females (*n* = 100)					
CC	13	28	Additive (CC vs. CA vs. AA)		0.03
CA	60	53			
AA	27	19			
AA	27	19	Homozygous (CC vs. AA)	0.33 (0.14–0.79)	0.01
CC	13	28			
CC	13	28	Heterozygous (CA vs. CC)	2.44 (1.15–5.19)	0.02
CA	60	53			
CC	13	28	Dominant (CA + AA vs. CC)	2.6 (1.26–5.39)	0.01
CA + AA	87	72			
CC + CA	73	81	Recessive (AA vs. CC + CA)	1.58 (0.81–3.07)	0.18
AA	27	19			
C	86	109	Allelic (A vs. C)	1.58 (1.07–2.36)	0.02
A	114	91			
All samples (*n* = 136)					
C	103	128	Allelic (A vs. C)	1.53 (1.06–2.2)	0.02
A	133	108			
rs5953283					
Males (*n* = 36)					
A	15	12	Allelic (G vs. A)	0.7 (0.27–1.83)	0.47
G	21	24			
Females (*n* = 100)					
AA	15	19	Additive (AA vs. AG vs. GG)		0.42
AG	39	44			
GG	46	37			
GG	46	37	Homozygous (AA vs. GG)	0.64 (0.28–1.42)	0.27
AA	15	19			
AA	15	19	Heterozygous (AG vs. AA)	1.12 (0.5–2.51)	0.78
AG	39	44			
AA	15	19	Dominant (AG + GG vs. AA)	1.32 (0.63–2.79)	0.45
AG + GG	85	81			
AA + AG	54	63	Recessive (AA vs. CC + CA)	1.45 (0.82–2.55)	0.2
GG	46	37			
A	69	82	Allelic (G vs. A)	1.32 (0.88–1.98)	0.18
G	131	118			
All samples (*n* = 136)					
A	84	94	Allelic (G vs. A)	1.2 (0.83–1.74)	0.34
G	152	142			

^1^ OR: odds ratio; 95% CI: 95% confidence interval.

**Table 2:** Methylation status of the studied CpG islands upstream the *FOXP3* promoter region in relation to *FOXP3* rs3761548 genotypes.

	*FOXP3* rs3761548 Genotypes							
Female	CC		AC		AA		Total	
Methylation status (positiveness found in heterozygosity +/−)	+	−	+	−	+	−	+	−
Patient (*n* = 15)	3	2	2	3	0	5	5	10
Control (*n* = 15)	0	5	0	5	0	5	0	15
							*p*-Value = 0.02	
Male	C		A				Total	
Methylation status	+		−				+	−
Patient (*n* = 10)	0	5	0	5			0	10
Control (*n* = 10)	0	5	0	5			0	10
							*p*-Value = 1.00	

**Table 3:** Primer sequences and conditions used in PCR amplification of *FOXP3* rs3761548 and *PPP1R3F* rs5953283 variants and amplification of CpG islands upstream from the promoter region of *FOXP3* for COBRA analysis.

Method	Primer Sequence (5′ → 3′)	PCR Conditions
PCR of rs3761548	F: CTTAACCAGACAGCGTAGAAGG R: CATCATCACCACGCTCTGG	95 °C for 5 min, 30 cycles of: 94 °C for 30 s, 55 °C for 30 s, 72 °C for 30 s 72 °C for 10 min
PCR of rs5953283	F: AGTCTCACTCTGTCACCTAGG R: GGTGTGATGATAGTATTGTGGGG	95 °C for 5 min, 30 cycles of: 94 °C for 45 s, 65 °C for 45 s (decreasing 0.5 °C/cycle), 72 °C for 1 min 72° for 10 min
For COBRA analysis	F: TTTTTTATTTTTTGGGTTTTTG R: AATAAAAAAAACAAAAACAAACAACTAA	94 °C for 5 min, 5 cycles of: 94 °C for 20 s, 60 °C for 20 s, 72 °C for 20 s 5 cycles of: 94 °C for 20 s, 58 °C for 20 s, 72 °C for 20 s 5 cycles of: 94 °C for 20 s, 56 °C for 20 s, 72 °C for 20 s 5 cycles of: 94 °C for 20 s, 54 °C for 20 s, 72 °C for 20 s 5 cycles of: 94 °C for 20 s, 52 °C for 20 s, 72 °C for 20 s 5 cycles of: 94 °C for 20 s, 51 °C for 20 s, 72 °C for 20 s 10 cycles of: 94 °C for 20 s, 50 °C for 20 s, 72 °C for 20 s 72 °C for 2 min

## Data Availability

The data presented in this study are available in this article.
