# Correction: Achilla et al. Genetic and Epigenetic Association of *FOXP3* with Papillary Thyroid Cancer Predisposition. *Int. J. Mol. Sci.* 2024, *25*, 7161

**DOI:** 10.3390/ijms26146725

**Published:** 2025-07-14

**Authors:** Charoula Achilla, Angeliki Chorti, Theodosios Papavramidis, Lefteris Angelis, Anthoula Chatzikyriakidou

**Affiliations:** 1 Laboratory of Medical Biology and Genetics, Faculty of Medicine, School of Health Sciences, Aristotle University of Thessaloniki, 54124 Thessaloniki, Greece; 2 First Propedeutic Department of Surgery, AHEPA University Hospital, Aristotle University of Thessaloniki, 54124 Thessaloniki, Greece; 3 School of Informatics, Aristotle University of Thessaloniki, 54124 Thessaloniki, Greece

In the original publication, there was a mistake in Table 3 as published [1]. The presented forward primer for the COBRA analysis was not the correct one used. The correct one is “F: TTTTTTATTTTTTGGGTTTTTG”. The scientific conclusions remain unaffected by this correction. The Academic Editor approved this correction. The original publication has also been updated. The corrected Table 3 and legend appear below.

## Figures and Tables

**Table 3:** Primer sequences and conditions used in PCR amplification of *FOXP3* rs3761548 and *PPP1R3F* rs5953283 variants and amplification of CpG islands upstream from the promoter region of *FOXP3* for COBRA analysis.

Method	Primer Sequence (5′ → 3′)	PCR Conditions
PCR of rs3761548	F: CTTAACCAGACAGCGTAGAAGG R: CATCATCACCACGCTCTGG	95 °C for 5 min, 30 cycles of: 94 °C for 30 s, 55 °C for 30 s, 72 °C for 30 s 72 °C for 10 min
PCR of rs5953283	F: AGTCTCACTCTGTCACCTAGG R: GGTGTGATGATAGTATTGTGGGG	95 °C for 5 min, 30 cycles of: 94 °C for 45 s, 65 °C for 45 s (decreasing 0.5 °C/cycle), 72 °C for 1 min 72° for 10 min
For COBRA analysis	F: TTTTTTATTTTTTGGGTTTTTG R: AATAAAAAAAACAAAAACAAACAACTAA	94 °C for 5 min, 5 cycles of: 94 °C for 20 s, 60 °C for 20 s, 72 °C for 20 s 5 cycles of: 94 °C for 20 s, 58 °C for 20 s, 72 °C for 20 s 5 cycles of: 94 °C for 20 s, 56 °C for 20 s, 72 °C for 20 s 5 cycles of: 94 °C for 20 s, 54 °C for 20 s, 72 °C for 20 s 5 cycles of: 94 °C for 20 s, 52 °C for 20 s, 72 °C for 20 s 5 cycles of: 94 °C for 20 s, 51 °C for 20 s, 72 °C for 20 s 10 cycles of: 94 °C for 20 s, 50 °C for 20 s, 72 °C for 20 s 72 °C for 2 min
